# Social Determinants Influencing Access to Home Delivery of Medication During the COVID-19 Pandemic for Cape Town Residents Living With Type 2 Diabetes

**DOI:** 10.1177/21501319251371807

**Published:** 2025-09-09

**Authors:** Klaus B. von Pressentin, Omotayo S. Alaofin, Graham Bresick, Neal David, Hayli Geffen, Natasha Moodaley, James Porter, Haniem Salie, Leigh Wagner, Robert J. Mash

**Affiliations:** 1 Division of Family Medicine, Department of Family, Community and Emergency Care, Faculty of Health Sciences, University of Cape Town, South Africa; 2 Division of Family Medicine and Primary Care, Department of Family and Emergency Medicine, Faculty of Medicine and Health Sciences, Stellenbosch University, South Africa; 3 Division of Epidemiology and Biostatistics, School of Public Health, Faculty of Health Sciences, University of Cape Town, South Africa

**Keywords:** COVID-19, type 2 diabetes, home delivery of medication, community health workers, health equity, primary healthcare, South Africa

## Abstract

**Objectives:**

The COVID-19 pandemic disrupted routine healthcare services, disproportionately affecting people living with chronic conditions such as type 2 diabetes (T2D). In response, the Western Cape Government Health implemented home delivery of medication (HDM) via community health workers (CHWs) to maintain continuity of care. This study aimed to evaluate the association between socioeconomic factors and access to HDM among T2D patients in Cape Town, South Africa, during the pandemic, with a focus on equity and health system responsiveness.

**Methods:**

A descriptive cross-sectional survey was conducted via telephone interviews with 267 patients receiving care at 4 public primary care facilities. Sociodemographic, economic, and treatment-related variables were collected. Fisher’s exact test and multivariable logistic regression were used to assess the associations between these variables and access to HDM.

**Results:**

Language, marital status, employment, access to piped water, distance from the clinic, and duration of diabetes were significantly associated with access to HDM. IsiXhosa-speaking and unmarried participants were less likely to receive HDM, while unemployed individuals and those with longer diabetes duration were more likely to benefit. Geographic and infrastructural barriers further limited access, suggesting that HDM implementation may have inadvertently excluded vulnerable groups.

**Conclusion:**

While HDM was a valuable innovation during the pandemic, its uneven reach highlights the persistence of health inequities. Language, social support, and geographic location emerged as key barriers. These findings underscore the need for inclusive, community-informed service design and the critical role of CHWs in delivering equitable, person-centred care. Future interventions should prioritise co-design with communities and address structural barriers to ensure equitable access to healthcare during crises and beyond.

## Background

Diabetes mellitus (DM) is a growing global health challenge, with prevalence projected to rise from 11.3% in 2030 to 12.2% by 2045, disproportionately affecting low- and middle-income countries (LMICs).^1^ In South Africa, the burden of type 2 diabetes (T2D) mirrors this trend, driven by rapid urbanisation, lifestyle changes, and rising obesity rates, particularly among women.^2,3^ The Western Cape Province, including Cape Town, carries a significant share of this burden, with high rates of diabetes-related morbidity and mortality.^4^

South Africa’s public health system has long grappled with the dual burden of communicable and non-communicable diseases (NCDs), compounded by deep-rooted socioeconomic inequities stemming from apartheid-era spatial and economic policies.^5^ These inequities continue to shape access to healthcare, particularly in historically marginalised communities such as those in the Cape Flats. Before the COVID-19 pandemic, efforts to address NCDs were being conceptualised within a community-oriented primary care (COPC) model that shifted perspective from the clinic to the community.^6^ The model included community-based services (CBS) delivered by community health worker (CHW) teams and nurse coordinators employed by local non-government organisations. This approach integrated primary care with public health and social support systems.^7^

The COVID-19 pandemic disrupted routine healthcare delivery, particularly for people living with chronic conditions.^8^ Lockdowns, mobility restrictions, and the reallocation of health system resources towards pandemic response disproportionately affected access to NCD services.^9,10^ In response, Western Cape Government Health (WCGH) leveraged its existing COPC model to implement home delivery of chronic medication (HDM) by CHWs, aiming to decongest facilities, protect vulnerable patients, and maintain facility continuity and medication adherence.^10-12^ This intervention was part of a broader strategy to ensure health system responsiveness and resilience during the crisis.^13^

However, implementing HDM services occurred within a complex web of social determinants.^13,14^ Language, housing, employment, and geographic location influenced who received care and who was left behind.^15^ These factors raised critical questions about equity of access to pandemic-era innovations and the extent to which health system adaptations were inclusive of the most vulnerable.

This study aimed to evaluate whether socioeconomic differences between patients determined the reach of this service innovation. In doing so, we consider the implementation of HDM not only as an adaptive response to COVID-19, but also as a lens through which to evaluate the health system’s capacity to deliver equitable, person-centred care under crisis conditions.

## Methods

### Study Design

This study employed a descriptive cross-sectional design.

### Study Setting

Four public sector primary care facilities were purposively selected, 1 from each of the 4 health substructures in Cape Town. These public sector facilities provided services to the indigent and uninsured majority of the population. Patients dependent on accessing care at these facilities were from low socioeconomic groups, ranging from informal settlements to formal low-cost housing. Care protocols for people with type 2 diabetes (T2D) were differentiated into stable and unstable groups based on their HbA1c levels (unstable was typically defined as greater than 8%) and the clinician’s judgment informed by evidence-based guidelines. Stable patients were seen every 6 months and received monthly pre-packaged medication via the pharmacy or alternative pick-up points. Unstable patients were seen every 1 to 2 months, and treatment was modified to try to achieve glycaemic control.

### Study Population

This paper forms part of a larger project that evaluated pandemic-specific health system interventions to reduce morbidity and mortality related to the COVID-19 pandemic among people living with diabetes in vulnerable communities in Cape Town, South Africa. For this larger project, a minimum sample of 200 was calculated to measure a 1% change in mean HbA1c over 1 year. The study population consisted of adult patients with T2D who had received care at 1 of the 4 selected primary care facilities for at least 6 months. Clinicians at these participating facilities assisted in generating lists of eligible patients returning to these facilities following the pandemic restrictions. The research team systematically selected every second patient from the clinician-generated lists for possible recruitment. The research team contacted these systematically selected patients telephonically and invited them to participate in the interview via a formal informed consent process.

### Study Instrument

The research team designed a structured, interviewer-administered questionnaire to collect data on HDM access, social demographics, economics, and treatment-related variables. Operational definitions included “formal housing” as a brick house or flat; “strong social support” as self-reported using the Oslo Social Support Scale (OSSS-3)^16^; and “access to piped water” referred to the availability of water inside the home versus in the yard or nearby.

### Data Collection

Three independent (not involved in service delivery at these facilities) and trained research assistants collected data between June and September 2021. The research assistants were fluent in all the local languages, particularly Afrikaans and isiXhosa. The research team designed and piloted an interviewer-administered questionnaire. The research assistants administered the questionnaire via telephone interview, taking 20 to 30 min per participant. The information provided was captured in the REDCap (Research Electronic Data Capture) database by the trained research assistants during the interviews. The REDCap data entry forms included built-in field validation rules (e.g. range checks and required fields) to ensure data completeness and consistency.

### Data Analysis

Data from REDCap were imported into the Statistical Package for Social Sciences (SPSS) version 28.0 (IBM Corporation, Armonk, New York, USA) and R version 4.3.0 (R Foundation, Vienna, Austria) for analysis by a statistician. Fisher’s exact test and multivariable logistic regression were used to assess the associations between sociodemographic, economic, and treatment-related variables and access to HDM. Participants who chose not to report their income were kept in the analysis as a separate group. This method was selected to maintain sample size and to prevent bias from excluding or imputing data. Variable categories (e.g. age, household size, distance to clinic) were selected based on the distribution of the data and considerations of statistical power. More detailed groupings were not possible due to small subgroup sizes.

### Ethical Considerations

The University of Cape Town Health Research Ethics Committee (HREC) approved the study (reference: 480/2020), and the Western Cape Government approved access to the 4 study sites (reference: WC_202009_013).

## Results

The study analysed the responses of 267 people living with T2D. The sociodemographic characteristics of the study participants are shown in Table 1. Most participants were female (64.8%), older than 50 years (71.9%), married (56.9%), and lived in households with more than 4 people (96.3%). Most (79.1%) spoke either Afrikaans or isiXhosa as their home language. There was a significant association (*P* < .001) between receiving HDM and being an older adult, as well as being married. Patients who spoke isiXhosa were significantly less likely (*P* < .001) to receive HDM. There was no significant association between gender, household size, or primary care facilities.

**Table 1.** Summary of Sociodemographic Variables Stratified According to Home Medication Delivery.

Variable	Home delivery of medication			*P*-value
	Overall (N = 267) n (%)	Yes (N = 171) n (%)	No (N = 96) n (%)	
Age group				<.001*
≤50	75 (28.1)	34 (19.9)	41 (42.7)	
>50	192 (71.9)	137 (80.1)	55 (53.7)	
Gender				.070
Male	94 (35.2)	67 (39.2)	27 (28.1)	
Female	173 (64.8)	104 (60.8)	69 (71.9)	
Language				<.001*
IsiXhosa	107 (40.1)	32 (18.7)	75 (78.1)	
Afrikaans	104 (39.0)	94 (55.0)	10 (10.4)	
Other	56 (21.0)	45 (26.3)	11 (11.5)	
Marital status				<.001*
Married	152 (56.9)	115 (67.3)	37 (38.5)	
Others	115 (43.1)	56 (32.7)	59 (61.5)	
Household size				.345
≤4	10 (3.7)	5 (2.9)	5 (5.2)	
>4	257 (96.3)	166 (97.1)	91 (94.8)	
Facility code				.743
Facility A	63 (23.6)	41 (24.0)	22 (22.9)	
Facility B	79 (29.6)	53 (31.0)	26 (27.1)	
Facility C	86 (32.2)	51 (29.8)	35 (36.5)	
Facility D	39 (14.6)	26 (15.2)	13 (13.5)	

* *p* is statistically significant (<.05).

Table 2 shows the socioeconomic characteristics of the patients with T2D. Most participants were unemployed (70.4%), had not completed high school (95.5%), earned less than $4,304 per year (70.4%), and relied on public transport (55.1%). A substantial minority lived in informal housing (20.2%) and did not have piped water in the home (17.2%). Most lived within 5 km of the primary care facility (57.7%). Over half of the patients (57.7%) reported strong social support and had access to a smartphone (79.4%).

**Table 2.** Summary of Socioeconomic Variables Stratified According to Home Delivery of Medication.

Variable	Home delivery of medication			*P*-value
	Overall (N = 267) n (%)	Yes (N = 171) n (%)	No (N = 96) n (%)	
Employment				.005*
Employed	79 (29.6)	40 (23.4)	39 (40.6)	
Unemployed	188 (70.4)	131 (76.6)	57 (59.4)	
Annual income				<.001*
None	7 (2.6)	6 (3.5)	1 (1.0)	
$0.06-$4304	181 (67.8)	140 (81.9)	(42.7)	
>$4304	8 (3.0)	4 (2.3)	4 (4.2)	
Declined to share	71 (26.6)	21 (12.3)	50 (52.1)	
Education				.222
Completed basic or secondary education	255 (95.5)	161 (94.2)	94 (97.9)	
Postsecondary education or technical training	12 (4.5)	10 (5.8)	2 (2.1)	
Transportation				.001*
Private	120 (44.9)	98 (57.3)	22 (22.9)	
Public transport	147 (55.1)	73 (42.7)	74 (77.1)	
Place of residence				<.001*
Brick house or flat	213 (79.8)	153 (89.5)	60 (62.5)	
Backyard dwelling	54 (20.2)	18 (10.5)	36 (37.5)	
Access to piped water				<.001*
In the house	216 (82.8)	159 (95.2)	57 (60.6)	
In the yard or nearby	45 (17.2)	8 (4.8)	37 (39.4)	
Access to electricity				.294
Yes	264 (98.9)	170 (99.4)	94 (97.9)	
No	3 (1.1)	1 (0.6)	2 (2.1)	
Distance from home to clinic				<.001*
Less than 5 km	154 (57.7)	118 (69.0)	36 (13.5)	
More than 5 km	113 (42.3)	53 (19.9)	60 (22.5)	
Access to a smartphone				<.001*
Yes	212 (79.4)	149 (87.1)	63 (65.6)	
No	55 (20.6)	22 (12.9)	33 (34.4)	
Social support				.592
Poor	22 (8.3)	15 (8.8)	7 (7.4)	
Moderate	90 (34.0)	54 (31.8)	36 (37.9)	
Strong	153 (57.7)	101 (59.4)	52 (54.7)	

* *p* is statistically significant (<.05).

Overall, 76.6% received HDM. Receiving HDM was significantly associated with being unemployed, having a lower annual income, having private transport, living in a formal structure with running water, residing closer to the facility, and having a smartphone (Table 2). Education, access to electricity, and social support were not significantly associated with HDM.

### Treatment-Related Factors

Table 3 presents diabetes and treatment-related factors associated with HDM. Most participants had lived with T2D for more than 5 years (71.5%), had access to a glucometer (58.4%), and nearly half were on both oral medication and insulin (46.8%). Having diabetes for longer and having access to a home glucometer were significantly associated with HDM.

**Table 3.** Summary of Treatment-Related Variables Stratified According to Home Delivery of Medication.

Variable	Home delivery of medication			*P*-value
	Overall (N = 267) n (%)	Yes (N = 171) n (%)	No (N = 96) n (%)	
Duration of diabetes				.001*
≤5 years	76 (28.5)	37 (21.6)	39 (40.6)	
>5 years	191 (71.5)	134 (78.4)	57 (59.4)	
Treatment				.614
Oral medication	113 (42.3)	69 (40.4)	44 (45.8)	
Insulin	29 (10.9)	18 (10.5)	11 (11.5)	
Both	125 (46.8)	84 (49.1)	41 (42.7)	
Access to a glucometer				.005*
Yes	156 (58.4)	111 (64.9)	45 (46.9)	
No	111 (41.6)	60 (35.1)	51 (53.1)	

* *p* is statistically significant (<.05).

Table 4 presents the results of the multivariable logistic regression analysis, which examines the association between predictors identified in the bivariable analysis (Tables 1-3) and the odds of having HDM. The odds of receiving HDM were reduced if you spoke isiXhosa (OR 0.10, 95% CI: 0.04-0.27), were not married (OR 0.27, 95% CI: 0.12-0.57), had piped water outside your home (OR 0.30, 95% CI: 0.10-0.84), and lived further away from the facility (OR: 0.42, 95% CI: 0.20-0.84). Conversely, the odds of receiving HDM were increased if you were unemployed (OR 2.50-fold, 95% CI: 1.14-5.58) or had T2D for longer (OR 2.23, 95% CI: 1.01-5.02). Age, transportation, smartphone, and glucometer access were not significantly associated with HDM.

**Table 4.** Results of Multivariable Logistic Regression Models Exploring the Association Between Predictors and Odds of Home Medication Delivery.

Variable	Panel	
Predictor	Adjusted OR (95% CI)	*P*-value
Age in years		
≤50	Ref	—
>50	1.52 (0.66-3.45)	.317
Language		
Afrikaans	Ref	—
IsiXhosa	0.10 (0.04-0.27)	<.001*
Other	0.71 (0.26-2.01)	.519
Marital status		
Married	Ref	—
Other	0.27 (0.12-0.57)	.001*
Employment		
Employed	Ref	—
Unemployed	2.50 (1.14-5.58)	.023*
Transportation		
Private	Ref	—
Public	1.87 (0.78-4.73)	.174
Access to piped water		
In your house	Ref	—
In your yard or nearby	0.30 (0.10-0.84)	.027*
Distance from home to clinic		
Less than 5 km	Ref	—
More than 5 km	0.42 (0.20-0.84)	.014*
Access to a smartphone		
No	Ref	—
Yes	1.73 (0.70-4.26)	.232
Duration of diabetes (years)		
≤5 years	Ref	—
>5 years	2.23 (1.01-5.02)	.049*
Access to a glucometer		
Yes	Ref	—
No	0.83 (0.40-1.73)	.618

* *p* is statistically significant (<.05).

## Discussion

### Summary of Key Findings

Our findings suggest that access to HDM was not equitable, raising questions about the service’s design and implementation. Speaking isiXhosa, being unmarried, being employed, not having piped water in the home, living further away from the facility, and having T2D for less than 5 years were associated with not receiving HDM. These results highlight how social determinants determined the reach of a pandemic-era service innovation intended to ensure adherence to medication and some continuity of care.

### Discussion of Key Findings

IsiXhosa-speaking participants were significantly less likely to receive HDM, despite no site-level differences in language distribution. This suggests that language may be a proxy for more profound systemic inequities, including socioeconomic status, housing, and access to smartphones or digital devices. Smartphone access was included in the model to adjust the association between language and HDM for smartphone access. The odds of smartphone access were reduced by a factor of 0.08 (92%, *P* < .001) for isiXhosa participants compared to Afrikaans participants. Although the type of housing is not in the model, the effect of language is essentially adjusted for it through piped water access (housing type is closely related to piped water access). Thus, the observed association between language and HDM accounts for factors potentially linked to language. The isiXhosa-speaking communities in Cape Town tend to occupy the lowest socioeconomic bands, with the highest level of informal dwellings and services. Language has been a barrier to accessing healthcare services.^17,18^

Similarly, being unmarried was associated with lower odds of receiving HDM. While marital status may reflect social support networks, it also intersects with gender norms, household structures, and caregiving roles. However, the level of social support did not show a significant association with HDM, suggesting that marital status may capture broader relational and structural dynamics. Prior studies have demonstrated that social support is crucial for effective diabetes self-management and emotional well-being.^19,20^

Unemployed participants were more likely to receive HDM, which is likely a reflection of logistical realities. Unemployed individuals are more likely to be home during delivery hours and are more reliant on public sector services. However, this finding also highlights the importance of considering how employment status intersects with access to care, particularly in contexts where informal work and precarious livelihoods are the norm. Other studies have suggested that access to primary care is challenging for employed individuals due to limited opening hours and a “no work, no pay” policy in many businesses.^21,22^

Geographic and infrastructural barriers also played a role. Participants living more than 5 km from the clinic or those without piped water inside their homes were significantly less likely to receive HDM. These findings underscore the structural determinants of health and the limitations of service delivery models that fail to account for spatial and infrastructural inequities fully. Community health workers may have faced logistical challenges in reaching certain areas, particularly informal settlements, where addresses were not well organised or where people lived in another catchment area.^13^ There may also have been safety issues for CHWs in the so-called “red zones” with high levels of crime and violence.^23,24^

Patients with a longer duration of diabetes were more likely to receive HDM, possibly reflecting their established relationships with the health facility and inclusion in chronic disease registries. This aligns with differentiated models of care, where stable patients are prioritised for decentralised services.^23,25^ However, it also raises concerns about excluding newly diagnosed or less-engaged patients from such innovations, especially if patient data is lacking or inaccurate in health information systems.^13,26^

These findings suggest that while HDM was a valuable intervention during the pandemic, its implementation may have inadvertently reinforced the impact of existing inequities.^27,28^ As noted in our reflections, receiving HDM serves as a proxy for evaluating the responsiveness and resilience of the health system. It also illustrates the tension between coordinating and integrating services, where attempts to ensure logistical efficiency may come at the cost of inclusivity.^11^

The role of CHWs in this intervention deserves particular attention. Their ability to deliver care under challenging conditions speaks to the flexibility of community-based primary care models. Yet, their effectiveness depends on adequate support, training, and integration into broader health system planning.^29^ The psychosocial toll on CHWs during the pandemic, including personal risk and community expectations, must also be acknowledged.^30^

This study contributes to the growing literature on pandemic-era innovations and their implications for equity in primary health care.^31-33^ It underscores the importance of designing interventions that are not only efficient but also inclusive and responsive to the lived realities of diverse patient populations. Nevertheless, the health system’s ability to pivot at scale and in a very short period to offer HDM suggests a high degree of resilience, agility, and innovation.

### Strengths and Limitations

It is essential to acknowledge that the study’s cross-sectional nature limits the ability to infer causality, and the reliance on self-reported data may introduce recall bias. Conducting telephone interviews with patients systematically identified by clinical staff returning to chronic care could introduce bias into the data collection and selection process, potentially excluding the most marginalised patients and those who were not contactable by phone. Research teams were unable to access facilities to oversee recruitment due to social distancing policies and research restrictions imposed during the COVID-19 pandemic. We were unable to verify self-reported access to HDM, which may also be subject to recall bias. The study, limited to 4 primary care facilities in Cape Town, may not represent the broader provincial or national settings. Furthermore, our findings may not be transferable to settings without established CHW networks or with different infrastructure. While the HDM model was feasible in Cape Town due to the existing COPC framework, its implementation in other settings would require adaptation to local health system structures and community dynamics. Finally, the study was conducted over several months during which HDM implementation may have evolved. This temporal variation could have influenced participant experiences and is noted as a limitation.

Despite the inherent limitations of a cross-sectional survey design, this study has several strengths. It provides valuable insights into the impact of socioeconomic factors on healthcare access during a critical period. Firstly, it gives a snapshot of the socioeconomic determinants influencing access to HDM during the COVID-19 pandemic among patients with T2D in Cape Town. Using a structured interviewer-administered questionnaire ensured consistency in data collection, and the telephonic method allowed for data gathering during a period when face-to-face interactions were limited. The study’s focus on a diverse sample from multiple primary care facilities across different Cape Town substructures enhances the relevance of the findings to similar urban settings.

### Recommendations

Many public health experts believe that it is a question of “when” rather than “if” we will face similar pandemics in the future, and this study points to ways in which we can be better prepared.^32^ Primary care facilities need to ensure that they have strong connections to the more marginalised and informal parts of the community in their catchment area. The COPC model of care, which links all households to CHW teams, can help address this issue, and ongoing implementation and scale-up are essential. Greater community engagement, through both formal and informal structures, can also increase trust, understanding, and connectivity.

Functional registration and empanelment systems with accurate addresses and contact details may enhance affiliation with the primary care facility. The value of this was highlighted during the COVID-19 pandemic, and such a system is planned for the rollout of national health insurance in South Africa. A specific primary care provider or facility would then know all patients.

Hiring CHWs who speak the local isiXhosa language, translating delivery instructions into other languages, and providing interpretation support to CHWs could help ensure that all patients fully comprehend and feel at ease with the HDM service. Future interventions should also accommodate the needs of employed workers by utilising technology such as e-lockers, which enable individuals to access medication at times and places convenient to them while maintaining physical distance during pandemics. These systems are being introduced in Cape Town as alternative pick-up points.

Future interventions must be co-designed with communities, informed by local data, and responsive to patients’ lived realities to promote equitable healthcare access. This study contributes to the broader discourse on health equity, offering a lens through which to evaluate the responsiveness of health systems during times of crisis and beyond.

## Conclusions

This study highlights how social determinants influenced HDM for people living with T2D in Cape Town during the COVID-19 pandemic. Key determinants such as language (used as a proxy for socioeconomic status), marital status, employment, geographic location, and duration of diabetes shaped who benefited from this service innovation. While HDM was a critical intervention to maintain medication adherence, its uneven reach underscores the persistent inequities embedded within the health system. The findings suggest that health systems can be better prepared to respond to crises such as a pandemic so that crisis adaptations are more equitably utilised. Fully implementing COPC, with a robust data management system tailored to the African context, and ensuring proper registration and empanelment of populations, while considering the challenges of access faced by employed workers, can contribute to this preparation.

## References

[1] International Diabetes Federation. IDF Diabetes Atlas. 10th edn. International Diabetes Federation; 2021. Accessed September 1, 2025. https://diabetesatlas.org/

[2] Sidahmed S Geyer S Beller J. Socioeconomic inequalities in diabetes prevalence: the case of South Africa between 2003 and 2016. BMC Public Health. 2023;23(1):324. doi:10.1186/s12889-023-15186-w 36788553 PMC9926686

[3] Sartorius B Veerman LJ Manyema M Chola L Hofman K. Determinants of obesity and associated population attributability, South Africa: empirical evidence from a national panel survey, 2008-2012. PLoS ONE. 2015;10(6):e0130218. doi:10.1371/journal.pone.0130218 PMC4463861 26061419

[4] Erasmus RT Soita DJ Hassan MS , et al. High prevalence of diabetes mellitus and metabolic syndrome in a South African coloured population: baseline data of a study in Bellville, Cape Town. S Afr Med J. 2012;102(11 Pt 1):841-844. doi:10.7196/samj.5670 23116739

[5] Ndinda C Ndhlovu TP Juma P Asiki G Kyobutungi C. The evolution of non-communicable diseases policies in post-apartheid South Africa. BMC Public Health. 2018;18(1):956. doi:10.1186/s12889-018-5832-8 30168397 PMC6117625

[6] Mash R Goliath C Mahomed H Reid S Hellenberg D Perez G. A framework for implementation of community-orientated primary care in the Metro Health Services, Cape Town, South Africa. Afr J Primary Health Care Fam Med. 2020;12(1):1-5. 10.4102/phcfm.v12i1.2632 PMC7756660 33354980

[7] National Department of Health, Republic of South Africa. National Strategic Plan for the Prevention and Control of Non-Communicable Diseases 2022 - 2027. National Department of Health, Republic of South Africa; 2022. Accessed September 1, 2025. https://www.health.gov.za/wp-content/uploads/2025/05/NCD-NSP-FINAL-VERSION-20-SEPT-22-1.pdf

[8] Bambra C Riordan R Ford J Matthews F. The COVID-19 pandemic and health inequalities. J Epidemiol Community Health. 2020;74(11):964-968. 32535550 10.1136/jech-2020-214401 PMC7298201

[9] Hofman K Levitt N Erzse A. Prioritising action on diabetes during COVID-19. S Afr Med J. 2020;110(8):719-720. doi:10.7196/SAMJ.2020.v110i8.14961 32880294

[10] Mash R Goliath C Perez G. Re-organising primary health care to respond to the Coronavirus epidemic in Cape Town, South Africa. Afr J Prim Health Care Fam Med. 2020;12(1):1-4. 10.4102/phcfm.v12i1.2607 PMC7669993 33181873

[11] Brey Z Mash R Goliath C Roman D. Home delivery of medication during coronavirus disease 2019, Cape Town, South Africa. Afr J Prim Health Care Fam Med. 2020;12(1):1-4. 10.4102/phcfm.v12i1.2449 PMC7284162 32501022

[12] David NJ Bresick G Moodaley N Von Pressentin KB. Measuring the impact of community-based interventions on type 2 diabetes control during the COVID-19 pandemic in Cape Town–a mixed methods study. S Afr Fam Pract. 2022;64(1):5558. 10.4102/safp.v64i1.5558 PMC9452916 36073102

[13] Mash RJ Schouw D Daviaud E Besada D Roman D. Evaluating the implementation of home delivery of medication by community health workers during the COVID-19 pandemic in Cape Town, South Africa: a convergent mixed methods study. BMC Health Serv Res. 2022/01/24 2022;22(1):98. doi:10.1186/s12913-022-07464-x 35073888 PMC8784590

[14] Mckenzie A Assegaai T Schneider H . South Africa: a primary health care case study in the context of the COVID-19 pandemic. 2023. Accessed May 30, 2025. https://iris.who.int/bitstream/handle/10665/372699/9789240061323-eng.pdf?sequence=1

[15] Arndt C Davies R Gabriel S , et al. Covid-19 lockdowns, income distribution, and food security: an analysis for South Africa. Glob Food Secur. 2020;26:100410. 10.1016/j.gfs.2020.100410 PMC7366977 32834955

[16] Azmiardi A Murti B Febrinasari RP Tamtomo DG. Low social support and risk for depression in people with type 2 diabetes mellitus: a systematic review and meta-analysis. J Prev Med Public Health. 2022;55(1):37-48. doi:10.3961/jpmph.21.490 35135047 PMC8841196

[17] Hill L Bekker S. Language, residential space and inequality in Cape Town: broad-brush profiles and trends. Afr Popul Stud. 2014;28:661. doi:10.11564/28-0-523

[18] Levin ME. Language as a barrier to care for Xhosa-speaking patients at a South African paediatric teaching hospital. S Afr Med J. 2006;96(10):1076-1079. 17164939

[19] Parviniannasab AM Faramarzian Z Hosseini SA Hamidizadeh S Bijani M. The effect of social support, diabetes management self-efficacy, and diabetes distress on resilience among patients with type 2 diabetes: a moderated mediation analysis. BMC Public Health. 2024;24(1):477. doi:10.1186/s12889-024-18022-x 38360647 PMC10868118

[20] Hasan AA Ismail A Noor H. The influence of social support on self-care behavior among T2DM patients. SAGE Open Nurs. 2024;10:23779608231219137. doi:10.1177/23779608231219137 PMC10768622 38186761

[21] Bresick GF Sayed A-R Le Grange C Bhagwan S Manga N Hellenberg D. Western Cape Primary Care Assessment Tool (PCAT) study: measuring primary care organisation and performance in the Western Cape Province, South Africa (2013). Afr J Prim Health Care Fam Med. 2016;8(1):e1-e12. doi:10.4102/phcfm.v8i1.1057 PMC4913443 27247157

[22] Govender K Girdwood S Letswalo D Long L Meyer-Rath G Miot J. Primary healthcare seeking behaviour of low-income patients across the public and private health sectors in South Africa. BMC Public Health. 2021;21(1):1649. doi:10.1186/s12889-021-11678-9 34503478 PMC8431853

[23] Mash R Christian C Chigwanda RV. Alternative mechanisms for delivery of medication in South Africa: a scoping review. S Afr Fam Pract. 2021;63(1):e1-e8. doi:10.4102/safp.v63i1.5274 PMC8424755 34476963

[24] Anstey Watkins J Griffiths F Goudge J . Community health workers’ efforts to build health system trust in marginalised communities: a qualitative study from South Africa. BMJ Open. 2021;11(5):e044065. doi:10.1136/bmjopen-2020-044065 PMC8137175 34011590

[25] Liu L Christie S Munsamy M , et al. Expansion of a national differentiated service delivery model to support people living with HIV and other chronic conditions in South Africa: a descriptive analysis. BMC Health Serv Res. 2021;21:1-8. 34001123 10.1186/s12913-021-06450-z PMC8127180

[26] Mutemaringa T Heekes A Smith M Boulle A Tiffin N. Record linkage for routinely collected health data in an African health information exchange. Int J Popul Data Sci. 2023;8(1):1771. 37636832 10.23889/ijpds.v8i1.1771 PMC10448229

[27] Tessema GA Kinfu Y Dachew BA , et al. The COVID-19 pandemic and healthcare systems in Africa: a scoping review of preparedness, impact and response. BMJ Glob Health. 2021;6(12):e007179. 10.1136/bmjgh-2021-007179 PMC8637314 34853031

[28] Pradhan NA Samnani AABA Abbas K Rizvi N. Resilience of primary healthcare system across low-and middle-income countries during COVID-19 pandemic: a scoping review. Health Res Policy Syst. 2023;21(1):98. 37723533 10.1186/s12961-023-01031-4 PMC10507852

[29] Mash B Ray S Essuman A Burgueño E. Community-orientated primary care: a scoping review of different models, and their effectiveness and feasibility in sub-Saharan Africa. BMJ Glob Health. 2019;4(8):e001489. 10.1136/bmjgh-2019-001489 PMC6703288 31478027

[30] Ndulue OI Chukka A Naslund JA. Burnout and mental distress among community health workers in low- and middle-income countries: a scoping review of studies during the COVID-19 pandemic. Glob Health J. 2024;8(4):162-171. doi:10.1016/j.glohj.2024.11.007

[31] Goodyear-Smith F Kidd M Oseni TIA , et al. International examples of primary care COVID-19 preparedness and response: a comparison of four countries. Fam Med Community Health. 2022;10(2):e001608. 10.1136/fmch-2022-001608 PMC9013790 35418499

[32] Wong WC-W Lin V Fang X Kidd M . The Lancet Commission on transforming primary health care in the post-COVID-19 era. Lancet. 2025;405:527-528. 39947214 10.1016/S0140-6736(25)00198-9

[33] Emanuel EJ Persad G Upshur R , et al. Fair allocation of scarce medical resources in the time of Covid-19. N Engl J Med. 2020;382(21):2049-2055. 32202722 10.1056/NEJMsb2005114

